# Trends, determinants and inequities of 4^+^ ANC utilisation in Bangladesh

**DOI:** 10.1186/s41043-016-0078-5

**Published:** 2017-01-13

**Authors:** Aminur Rahman, Monjura Khatun Nisha, Tahmina Begum, Sayem Ahmed, Nurul Alam, Iqbal Anwar

**Affiliations:** 1 International Centre for Diarrhoeal Disease Research, Dhaka, Bangladesh; 2 University of Sydney, Sydney, Australia

**Keywords:** Antenatal care, Trends, Determinants, Inequality, Bangladesh

## Abstract

**Background:**

The objectives of this study are to document the trend on utilisation of four or more (4^+^) antenatal care (ANC) over the last 22 years period and to explore the determinants and inequity of 4^+^ ANC utilisation as reported by the last two Bangladesh Demographic and Health surveys (BDHS) (2011 and 2014).

**Methods:**

The data related to ANC have been extracted from the BDHS data set which is available online as an open source. STATA 13 software was used for organising and analysing the data. The outcome variable considered for this study was utilisation of 4^+^ ANC. Trends of 4^+^ ANC were measured in percentage and predictors for 4^+^ ANC were measured through bivariate and multivariable analysis. The concentration index was estimated for assessing inequity in 4^+^ ANC utilisation.

**Results:**

Utilisation of 4^+^ ANC has increased by about 26% between the year 1994 and 2014. Higher level of education, residing in urban region and richest wealth quintile were found to be significant predictors. The utilisation of 4^+^ ANC has decreased with increasing parity and maternal age. The inequity indices showed consistent inequities in 4^+^ ANC utilisation, and such inequities were increased between 2011 and 2014.

**Conclusions:**

In Bangladesh, the utilisation of any ANC rose steadily between 1994 and 2014, but progress in terms of 4^+^ ANC utilisation was much slower as the expectation was to achieve the national set target (50%: 4^+^ ANC utilisation) by 2016. Socio-economic inequities were observed in groups that failed to attend a 4^+^ ANC visit. Policymakers should pay special attention to increase the 4^+^ ANC coverage where this study can facilitate to identify the target groups whom need to be intervened on priority basis.

## Background

Although Bangladesh has made a remarkable progress in achieving the targets for Millennium Development Goals (MDGs) 4 and 5, maternal mortality ratio (MMR) (170 per 100,000 live births) [1] and neonatal mortality rate (NMR) (28 per 1000 live births) [2] are still considerably high. One of the major reasons for these high mortality rates is low utilisation of maternal health services such as antenatal care (ANC), skilled birth attendance (SBA) at delivery and postnatal care (PNC) [3].

ANC by a medically trained provider is a fundamental determinant for ensuring safe delivery practices that plays an important role to prevent, detect and treat the women from fetal loss or complications related to pregnancy. Evidence suggests that good quality ANC can reduce maternal and perinatal morbidity and mortality [4] by identifying women at risk of suffering anaemia, pregnancy induced hypertension and preterm labour [5–7]. It is well documented that ANC use has a protective impact on the pregnancy outcomes including low birth weight and preterm births [6–8]. ANC educates pregnant women about the warning signs and symptoms of birth spacing and family planning issues [9]. Also, evidence shows that ANC increases the use of a SBA (doctor, nurse or midwife) during delivery and postnatal care [10].

However, certain predictors ranged from health system to user’s attributes are responsible for ANC utilisation particularly in developing countries like Bangladesh. These are availability, accessibility, quality of ANC, women’s socio-economic status, demographic factors, women’s education, perceived knowledge, cultural beliefs, and previous obstetric history [11–13].

The quality of ANC is measured by three dimensions—number of visits, timing of initiation of care and inclusion of all recommended components of care [14]. Total number of ANC visits has a significant impact on a number of pregnancy outcomes [15, 16]. A study from Bangladesh found that women who had one or no ANC visit were two times more likely to suffer from a perinatal death compared with women who had three or more ANC visits [17].

The World Health Organization (WHO) recommends that every pregnant woman should have a minimum of four focused antenatal visits [18]. Though the importance of this recommended four focused ANC visits and its positive effects are already documented by several studies [19–21], the proportion of the women utilising four or more (4^+^) ANC is not still at satisfactory level. Globally, only about 55% of pregnant women utilised the recommended minimum four times antenatal care during the period 2005–2012. If we look at only the low-income countries in the same period, the situation is much worse (37%) [18].

The recent Bangladesh Demographic and Health Survey (BDHS) 2014 data has reported that the majority of the pregnant women (64%) in Bangladesh receive ANC from the medically trained providers, such as qualified doctor, nurse, midwife or paramedic, family welfare visitor (FWV), community skilled birth attendant (CSBA), medical assistant (MA) or sub-assistant community medical officer (SACMO). The facilities which provide ANC services can vary in Bangladesh, such as community clinic and family welfare center at union level, upazila health complexes at sub-district level and district and tertiary hospitals at district or national level. Moreover, private doctors’ chambers and NGO clinics also provide ANC services. Though the utilisation of at least one ANC was around 70%, women attending for WHO-recommended 4^+^ ANC was very less (31%) during that period [3]. These data suggest that Bangladesh lags behind in reaching the national target of 50% 4^+^ utilisation by the year 2016. Furthermore, less research has been done to identify the determinants of 4^+^ utilisation in particular, though several of those were looking at the determinants of ANC utilisation in Bangladesh in general [22]. Thus, to address this gap and to meet the target of Sustainable Development Goal (SDG) 3 of increasing 4^+^ ANC coverage to 98% by 2030 [23], the country needs a comprehensive strategy and specific milestone.

The present study attempts to identify the determinants of utilisation and inequity of 4^+^ ANC utilisation in order to offer insights to policymakers about the different public health strategies to increase the coverage of 4^+^ ANC in Bangladesh.

## Methods

### Data source

This paper has used the electronically available BDHS data from 2011 to 2014 surveys to look at the determinants of 4^+^ ANC, inequity in utilisation. The study also reported the trends of 4^+^ ANC utilisation from all seven surveys. Data were retrieved from the MEASURE DHS (Monitoring and Evaluation to Assess and Use Results Demographic and Health Surveys) website www.measuredhs.com. In total, seven DHSs have been conducted over the last 22 years period: 1994, 1997, 2000, 2004, 2007, 2011 and 2014. BDHS is a nationally representative sample survey which provides the information on basic demographic and health indicators including fertility, contraceptive knowledge and use, maternal and child health, nutritional status of mothers and children, awareness of AIDS and domestic violence.

### Study population

Women who gave their last birth in the preceding 3 years of each survey were included as study participants.

### Outcome variable

Women who utilised 4^+^ ANC in their last pregnancy were considered as outcome variable. The outcome variable was recoded as a binary variable (yes/no) in the merged dataset.

### Explanatory variable

A set of socio-demographic variables related to the utilisation of ANC were identified from the survey data, such as maternal age in years, women education, parity, residence of participants both by administrative divisions and by area, religion and wealth quintile. The household economy represented as wealth quintile were calculated separately from the available wealth variables such as housing materials, type of latrines, availability of electricity, and ownership of radio and/or television for each survey year. Principal component and factor analysis statistical method proposed by Filmer and Gwatkin in their study was used to compute the wealth quintile for this study [22, 24, 25].

### Data analysis

Trends of 4^**+**^ ANC utilisation were calculated from frequency percentages by the survey years to observe the changes over time. Also, frequency tabulations were generated to describe women’s socio-demographic characteristics and their distributions over two-survey periods 2011 and 2014, which were compared by using a chi-squared test. We performed binary logistic regression to obtain both crude and adjusted odds ratio (OR) for our outcome variable using the Wald test to assess the statistical significance at 95% confidence intervals (CI). The analysis was adjusted for all the other variables which might have any confounding effect. Stata version 13 was used for analysing data.

Inequities in utilisation of 4^**+**^ ANC were estimated across wealth quintiles. We estimated concentration index as a relative measure among the subgroups of population. concentration index was estimated using the concentration curve. The concentration curve represents the cumulative proportion of 4^**+**^ ANC utilisation ranked by socio-economic quintiles of the households against cumulative proportions of population in the corresponding households. Then the concentration index is the difference in twice the area between the concentration curve and the diagonal [26–28]. The concentration index can range between −1 and +1, where 0 implies perfect equity, a positive value implies 4^**+**^ ANC utilisation is more concentrated among the better off and negative value implies such visits are more concentrated among the less affluent [27, 29].

## Results

A total of 16,889 ever married women of reproductive age, who had their last birth in the 3 years preceding the survey were included in the last two BDHS, where BDHS 2011 and BDHS 2014 included 8798 and 8091 women, respectively (Table 1). Of those, only 1323 women in BDHS 2011 and 1435 women in BDHS 2014 utilised 4^+^ ANC (Table 2).

**Table 1:** Socio-demographic characteristics of the women surveyed in BDHS 2011 and 2014

Variables	Survey year		*P* value
	2011 (*N* = 8798)	2014 (*N* = 8091)	
	*n* (%)	*n* (%)	
Age (Years)			
≤19	1207 (13.7)	1185 (14.6)	0.00
20–29	5628 (64.0)	4991 (61.7)	
30–39	1727 (19.6)	1770 (21.9)	
≥40	236 (2.7)	145 (1.8)	
Education			
No education	1785 (20.3)	1328 (16.4)	0.00
Primary	2712 (30.8)	2264 (28.0)	
Secondary	2686 (41.9)	3746 (46.3)	
Higher	615 (7.0)	754 (9.3)	
Parity			
1	2465 (28.0)	3133 (38.7)	0.00
2	2801 (31.8)	2426 (30.0)	
3	1708 (19.4)	1301 (16.1)	
4	896 (10.2)	641 (7.9)	
5	437 (5.0)	289 (3.6)	
≥6	490 (5.6)	300 (3.7)	
Division			
Barisal	491 (5.6)	458 (5.7)	0.00
Chittagong	2018 (22.9)	1748 (21.6)	
Dhaka	2732 (31.1)	2831 (35.0)	
Khulna	796 (9.0)	611 (7.6)	
Rajshahi	2078 (23.6)	1626 (20.1)	
Sylhet	682 (7.8)	818 (10.1)	
Religion			
Muslim	8063 (91.6)	7407 (91.5)	0.79
Hindu and others	735 (8.4)	685 (8.5)	
Area			
Rural	6840 (77.7)	6036 (74.6)	0.00
Urban	1958 (22.3)	2056 (25.4)	
Wealth quintile			
Poorest	1033 (11.7)	1837 (22.7)	0.00
2nd quintile	1255 (14.3)	1549 (19.1)	
3rd quintile	1698 (19.3)	1566 (19.4)	
4th quintile	1954 (22.2)	1603 (19.8)	
Richest	2858 (32.5)	1536 (19.0)

**Table 2:** Factors associated with 4^+^ ANC utilisation in Bangladesh; BDHS (2011 and 2014)

Predictors	2011		2014	
	4^+^ ANC (*N* = 1323)	Adjusted OR (95% CI)	4^+^ ANC (*N* = 1435)	Adjusted OR (95% CI)
	*n* (%)		*n* (%)	
Age				
≤19	263 (19.9)	1.00	281 (19.6)	1.00
20–29	837 (63.3)	1.22 (1.00–1.48)	901 (62.8)	1.19 (0.97–1.45)
30–39	215 (16.3)	1.79 (1.31–2.44)	245 (17.1)	1.39 (1.03–1.86)
≥40	8 (0.6)	1.12 (0.47–2.67)	8 (0.6)	0.76 (0.32–1.83)
Education				
No education	80 (6.0)	1.00	87 (6.1)	1.00
Primary	243 (18.4)	1.47 (1.11–1.95)	260 (18.1)	1.34 (1.01–1.77)
Secondary	723 (54.6)	2.74 (2.09–3.60)	763 (53.2)	2.00 (1.52–2.62)
Higher	277 (20.9)	6.83 (4.83–9.66)	325 (22.6)	3.62 (2.60–5.06)
Parity				
1	625 (47.2)	1.00	686 (47.8)	1.00
2	420 (31.7)	0.78 (0.65–0.94)	466 (32.5)	0.88 (0.74–1.06)
3	191 (14.4)	0.72 (0.56–0.91)	179 (12.5)	0.73 (0.57–0.93)
4	61 (4.6)	0.49 (0.34–0.69)	60 (4.2)	0.59 (0.42–0.85)
5	15 (1.1)	0.26 (0.14–0.47)	32 (2.2)	0.80 (0.50–1.29)
≥6	11 (0.8)	0.22 (0.11–0.43)	12 (0.8)	0.38 (0.19–0.74)
Division				
Sylhet	157 (11.9)	1.00	138 (9.6)	1.00
Barisal	217 (16.4)	1.49 (1.11–2.01)	243 (16.9)	1.15 (0.86–1.54)
Chittagong	207 (15.6)	1.02 (0.78–1.33)	286 (19.9)	1.07 (0.83–1.39)
Dhaka	208 (15.7)	1.21 (0.92–1.59)	217 (15.1)	1.37 (1.06–1.77)
Khulna	396 (29.9)	1.69 (1.28–2.24)	404 (28.2)	2.00 (1.52–2.63)
Rajshahi	138 (10.4)	1.93 (1.51–2.48)	147 (10.2)	1.95 (1.53–2.49)
Area				
Rural	657 (49.7)	1.00	772 (53.8)	1.00
Urban	666 (50.3)	2.14 (1.84–2.5)	663 (46.2)	1.46 (1.24–1.7)
Wealth quintile				
Poorest	76 (5.7)	1.00	151 (10.5)	1.00
2nd quintile	100 (7.6)	1.06 (0.75–1.49)	181 (12.6)	1.21 (0.95–1.56)
3rd quintile	134 (10.1)	0.90 (0.65–1.24)	222 (15.5)	1.33 (1.04–1.70)
4th quintile	270 (20.4)	1.37 (1.01–1.84)	367 (25.6)	2.14 (1.68–2.72)
Richest	743 (56.2)	1.82 (1.36–2.43)	514 (35.8)	3.57 (2.73–4.68)

### Trends of 4^+^ ANC utilisation

**Fig. 1:**
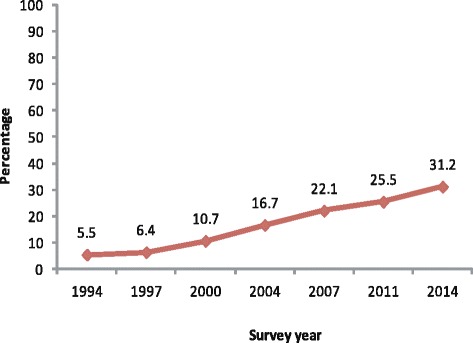
Trend in attending 4^+^ antenatal care services (1994–2014)

The utilisation of 4^**+**^ ANC was relatively less though there was a rising trend observed over the years. Overall, the utilisation of 4^**+**^ ANC rose by about 25.7% over 22 years (Fig. 1). In 1994, the proportion of utilising 4^**+**^ ANC was 5.5% among the women who had their last birth in 3 years preceding the survey which increased to 31.2% in 2014. From the year 1994 to 1997, the progress was much slower regarding the utilisation of 4^**+**^ ANC, while between the years 1997 and 2014 the increase was about 24.8%.

#### Socio-demographic characteristics of the women surveyed under BDHS 2011 and 2014

The overall characteristics of study population are tabulated in Table 1.

The majority (two-thirds) of the maternal age group were attributable to 20–29 years and more than one third of study population had attained secondary level of education in both the surveys. The percentage of women having no education reduced by about 4% (20.3% in 2011 and 16.4% in 2014) and higher level of education increased by about 2.5% (7% in 2011 and 9.3% in 2014) in 2014 than in 2011. The proportion of multiparous women decreased significantly in the year 2014 compared to 2011. However, substantial regional variation in participation in the survey has also been observed. Dhaka division had the highest proportion of women attending the survey while the lowest proportion of women belonged to the Barisal division. Still, two-thirds of the participants were the inhabitants of a rural region in both the surveys. Considering religion, around 91% women were Muslims. The quintile distribution of the participants was three times higher in the richest quintile than the poorest (32.5 vs. 11.7%) in 2011, but in 2014 there is less variation observed. During 2011, the lowest two quintile distributions were less than 15% and in the richest group, it was over 30%.

### Socio-demographic determinants of 4^+^ ANC utilisation (2011–2014)

Among all the selected socio-demographic variables, women aged 30–39 years age group, increased level of education, residing in the urban area and the richest wealth quintile were positively associated with the 4^+^ ANC utilisation (Table 2).

Proportion of utilising 4^+^ ANC showed a tendency to decrease with the increasing number of parity in both the surveys. Women having more kids were less likely to attend 4^+^ ANC than the women who was pregnant for the first time.

It was observed in both the surveys that the women with increased level of education were more likely to utilise the 4^**+**^ ANC than the women who had no formal education. Furthermore, the likelihood of utilising 4^**+**^ ANC was about two times more among the urban dwellers than their rural counterparts during both the surveys. The rich-poor differences on utilising 4^+^ ANC were still prominent in between two-survey years. The richest are 1.82 times (CI: 1.36–2.43; *P* < 0.005) and 3.6 times (CI: 2.73–4.68; *P* < 0.005) more likely to utilise the 4^**+**^ ANC services than the poorest group in BDHS 2011 and 2014, respectively. The administrative division also had an influencing contribution for 4^**+**^ ANC utilisation. The likelihood of those utilising 4^+^ ANC had increased in all administrative divisions compared to those residing in the Sylhet division where the Khulna and Rajshahi division came as significant in both the survey years (*P* < 0.005).

#### Wealth-related inequity of 4^+^ ANC utilisation

**Fig. 2:**
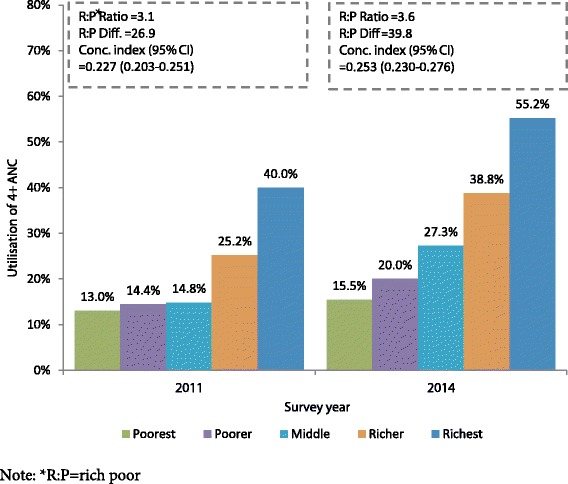
Inequities in 4^+^ ANC utilisation across wealth quintiles over survey years

The concentration index indicated existence of wealth-related inequity in 4^+^ ANC utilisation in the year 2011 and 2014. However, such inequities were increased between 2011 and 2014 (Fig. 2). The values of concentration indices for 4^+^ ANC utilisation were increased from 0.227 to 0.253 between these periods.

## Discussion

This study has identified that the WHO-recommended 4^**+**^ ANC utilisation is on an upward trend in Bangladesh, from the initial rate of 5.5% in 1994 to 31.2% in 2014 BDHS. However, this figure implies that still two-thirds do not go for 4^**+**^ ANC services comparing the national target of 50% mentioned in the third 5-year sector program Health, Population and Nutrition Sector Development Program 2011–2016. The low percentage of 4^**+**^ ANC utilisation has also been documented in other developing countries and postulated that late initiation of the first ANC could be the contributor of such low attendance [16, 30, 31]. On the contrary, 4^**+**^ ANC utilisation during pregnancy has already been considered as a policy priority as a means to ensure high quality ANC and delivery care in many of the countries across the world [30, 32]. For example, Bangladesh and Tanzania have already been shifted to FANC (focused antenatal care) with 4^**+**^ ANC and the first ANC before 4 months of gestational age [32]. However, little is known about the determinants of 4^+^ ANC utilisation particularly in a Bangladesh perspective.

Against this backdrop, the present study has attempted to explore the contributing factors of 4^**+**^ ANC utilisation. The present study found that pregnant women having higher education were four to seven times more likely to utilise 4^+^ ANC than those women with no formal education. This finding has similarity with the study done in Indonesia and some other countries having similar context [13, 32]. Another study which was done to identify the determinants of any ANC attendance in rural Bangladesh has also identified female education as the positive contributor [13]. Female education implies women empowerment where she is more mindful about using health services with expected increase level of financial and geographical access [33]. Moreover, one study form Nepal also recommended female education as a policy priority to boost ANC utilisation [30].

The geographical variation of 4^+^ ANC utilisation has also been observed in this study where those residing in the Rajshahi and Khulna division showed better picture in terms of 4^+^ ANC utilisation. Dhaka being the capital city has reported more proportion of women utilising 4^+^ ANC than Sylhet but did not reveal any statistically significant association. Thus, further evaluation is required to identify the factors related to this hindrance. However, reported high fertility rate, low female literacy rate and comparatively having higher hard-to-reach area could be considered as the main reasons for low level of 4^+^ ANC utilisation for the Sylhet division. So, it was considered as a reference category to compare how better the situation of other divisions are. Since city dwellers are blessed with good transport communication, they could have better access to the health facility. This in turn reported as the positive contributor of 4 ^+^ ANC utilisation among the women residing in larger cities and divisions in other parts of the world like Tanzania [32].

Younger and less parous women were more likely to utilise 4^**+**^ ANC among this study population which is more in line with other research findings from the developing countries [12, 34]. Younger women are less experienced on birthing matters for which they seek more prenatal visit [32]. On the contrary, a mother having one or two children at home may get less chance to think about her own health and the unseen baby in her womb rather keeping busy with kids rearing and additional household responsibilities [35]. Moreover, higher prevalence of 4^**+**^ ANC utilisation among 20–29 years of age group can be better explained by the predominance of child bearing at these ages in a Bangladesh context [2].

The result showed significant socio-economic inequity exists in utilisation of 4^+^ ANC in both years. Therefore, the economically better off mothers were utilising 4^+^ ANC more compared to poor mothers. The concentration index also showed wealth-related inequity in 4^**+**^ ANC utilisation were higher in 2014 (Concentration index = 0.253) compared to 2011 (Concentration index = 0.227). Earlier studies showed such inequity in overall utilisation of ANC was decreased over time (1994–2011) [36–39]. To understand the reason behind this slight increment of inequity between 2011 and 2014 further decomposition analysis of inequity is required. Bangladesh has already been entitled as a nation providing “Good health services at low cost” even though majority of the poor women failed to utilise recommended numbers of 4^+^ ANC [40].

## Conclusions

In Bangladesh, the utilisation of any ANC rose steadily between 1994 and 2014. However, 4^+^ ANC progress was much slower than expectation though having documented several strong benefits on maternal health outcome. Our findings indicate that wealthier and more educated women, as well as those living in urban areas, are the major users of 4^+^ ANC, in Bangladesh. Thus, priority focus should be given to implementing and evaluating interventions that benefit women who are poorer, less educated and living in rural areas. Providing 4^+^ ANC irrespective of economic status and residence of pregnant women could ensure universal maternal health coverage as committed on a national strategic guideline [41].

### Strength and limitation

The strength of the study is that it is using the publicly available national demographic data which has less missing information and is more accurate to interpret. The inference made from this study is representative of whole nation. The only limitation is that women’s own perception and local context could not be answered through this quantitative data for which further qualitative exploration is needed.

## Abbreviations

4^+^ ANC: 4 or more Ante Natal Care
BDHS: Bangladesh Demographic and Health Survey
C-section: Caesarean section
FD: Facility delivery
MEASURE DHS: Monitoring and Evaluation to Assess and Use Results Demographic and Health Surveys
NIPORT: National Institute of Population Research and Training
SBA: Skilled Birth Attendant
